# Why Pulmonary Vasodilation May Be Part of a Key Strategy to Improve Survival in COVID-19

**DOI:** 10.7759/cureus.20746

**Published:** 2021-12-27

**Authors:** Isaac Solaimanzadeh

**Affiliations:** 1 Internal Medicine, Interfaith Medical Center, New York, USA

**Keywords:** covid-19, ventilation perfusion mismatch, pulmonary vasodilation, hypoxia, coronavirus disease, calcium channel blockers, hypoxic pulmonary vascoconstriction

## Abstract

Oxygenation is a function of both ventilation and perfusion. While approaches to the treatment of COVID-19 have focused largely on ventilation strategies and antiviral therapies, attention towards the improvement of vascular perfusion defects has been neglected. This article examines clinical findings that indicate perfusion defects are a critical component of COVID-19 pathophysiology. They also support the notion that medications that promote perfusion with pulmonary vasodilatation can yield significantly improved outcomes that include overall survival.

Calcium channel blocker usage has been associated with improved survival and outcomes in several retrospective reviews of patient populations with COVID-19 from across the world. This includes studies conducted in Paris, France; Wuhan, China; Daegu, South Korea; Brooklyn, New York; Brussels, Belgium; and a national sample from across the United States*. *These medications are generally prescribed to treat hypertension. Yet, they are also utilized in various pulmonary conditions to effectuate pulmonary vasodilatation. Thus, a concomitant benefit appears to have been revealed as patients that were taking these medications had significantly improved overall survival. Sildenafil is another medication that induces pulmonary vasodilatation. It was found to decrease the need for mechanical ventilation and reduce hospital length of stay in COVID-19 in a triple-blinded randomized control trial.

The importance of pulmonary vasodilation in COVID-19 has been evaluated further. In a study of over 100 high-resolution CT scans, patients with COVID-19 showed a significant reduction in pulmonary blood volume contained in small blood vessels of <5 mm^2^ compared to healthy volunteers. Moreover, this was found to clinically correlate with a need for more oxygen supplementation. In radiologic perfusion studies, hypoperfusion was observed to occur in the healthy lung while hyperperfusion was present in non-healthy COVID-inflicted lung. It appears that perfusion of oxygen-carrying capacity, in the form of hemoglobin-carrying red blood cells, is being misappropriated towards unhealthy lung tissue. This was observed concurrently while the healthy lung had a paucity of perfusion. This can be a key aspect of hypoxic development in COVID-19. Mathematical modeling of perfusion abnormalities in COVID-19 has also implicated extensive perfusion defects, with ventilation-perfusion mismatching in the non-injured lung and hyperperfusion of up to threefold increases to afflicted regions. Vasodilation in the form of systemic intravascular medications may help improve outcomes by resetting this imbalance and by promoting perfusion of the alveolar-capillary unit where gas exchange and oxygenation occurs particularly in the non-injured lung.

Furthermore, endothelialitis and microthrombosis have been observed on pathology specimens as many patients develop micro-thrombi following prolonged perfusion deficits. Vasodilatory agents can curb vasoconstriction and drive more perfusion towards healthy tissue. The temporal matching of consistent systemic intravascular vasodilation therapy throughout the gradual and progressive course of the illness may be integral to achieving improved outcomes. Improving perfusion to healthy tissue can help improve oxygenation and overall outcomes in COVID-19. These findings support further utilization and investigation of vasodilatory agents in the treatment of COVID-19.

## Introduction

Hypoxia may develop in the context of ventilation-perfusion mismatch. Both elements, namely, ventilation and perfusion are necessary components to maintain adequate oxygenation. Approaches to the treatment of COVID-19 have focused exceedingly on ventilation strategies and antiviral therapies. Yet, attempts at the improvement of vascular perfusion defects have received less attention. This article serves to describe pertinent aspects related to perfusion abnormalities in COVID-19. It also attempts to highlight the value of medications capable of counteracting vasoconstriction that effectively augments perfusion. The clinical studies reviewed reveal the potential benefits of this method to improve outcomes including overall survival. 

## Materials and methods

This article will consider relevant literature to better understand how and why pulmonary vasodilation can be an effective strategy to help patients survive COVID-19. It will do so by examining articles that provide insight into the pathophysiologic processes of this disease. Information is gathered from various sources including advanced radiologic imaging, and pathology specimens.

In addition, medications known to affect pulmonary vasodilation that was administered to patient populations, are reflected upon. These include calcium channel blocking agents as well as phosphodiesterase inhibition. Articles include clinical reviews from across the world. Radiologic studies go past standard imaging utilized in clinical management at the bedside and peer into high-resolution imaging that offers deeper insight into pathologic processes. Furthermore, iodine enhanced perfusion CT scans are also underscored. Surveys of these radiologic studies include actual patients suffering from the disease as well as those subject to oxygen supplementation. Altogether, integration of retrospective reviews of medications, clinical findings as well as radiologic and pathology specimens are all called upon to better understand the role of pulmonary vasodilation in COVID-19.

## Results

To begin with, calcium channel blockers (CCBs) were found to improve survival and outcomes in retrospective studies of patients suffering from COVID-19 across the world. This includes studies conducted in Paris, France [1]; Wuhan, China [2]; Daegu, South Korea [3]; Brooklyn, New York [4]; Brussels, Belgium [5]; and across the United States [6] as seen in Table 1.

**Table 1:** The use of calcium channel blockers reveals improved outcomes from various retrospective studies across the world CCBs: Calcium channel blockers, OR: Odds ratio, CI: Confidence interval

Location	Number of Patients	Findings	Calcium Channel Blocker(s)	Data
Paris, France (Neuraz et al. [1])	3,965	Significantly decreased in-hospital mortality in patients with COVID-19 infection.	Amlodipine, Diltiazem, Felodipine, Isradipine, Lacidipine, Lercanidipine, Manidipine, Nicardipine, Nifedipine, Nitrendipine, Verapamil.	Statistically significant reduction of the risk of death; Natural Language Processing Hazard Ratio: 0.82, 95% CI: 0.71-0.94; p=0.005.
Wuhan, Hubei, China (Zhang et al. [2])	225	Significantly reduced case fatality rate and risk of death with amlodipine use.	Amlodipine.	Case fatality rate significantly decreased from 19.5% (15/77) in non-amlodipine besylate-treated group to zero (0/19) in amlodipine besylate-treated group (p= 0.037).
Daegu, South Korea (Kim et al. [3])	1,374,381	Significantly lower risk of COVID-19.	Calcium channel blockers; unspecified.	Significantly lower risk of COVID-19 for those prescribed CCBs and compliant (Relative risk 0.768; 95% CI: 0.601–0.980).
Brooklyn, New York, USA (Solaimanzadeh [4])	65	Significantly improved survival and decreased risk of intubation.	Nifedipine, Amlodipine.	Significantly more likely to survive: 50% CCB vs. 14.6% No-CCB (P < .01 ; p=0.0036) Significantly less likely to be intubated 4.2% CCB vs. 39.0% No-CCB (P < .01, p=0.0026).
Brussels, Belgium (Darquennes et al. [5])	317	Significantly decreasing odds ratio of in-hospital death associated with long-term treatment with amlodipine.	Amlodipine.	Decreased odds ratio of in-hospital death associated with long-term treatment (OR 0.24, 95% CI: 0.09–0.62; p=0.0031).
United States (National Sample) (Rosenthal et al. [6])	64,781	Significantly decreased odds of mortality.	Calcium channel blockers; unspecified.	Decreased odds of death (OR 0.73; 95% CI: 0.68-0.79; P < .001).

The use of CCBs in elderly (>65 years old) hospitalized patients with COVID-19 was significantly associated with improved survival and decreased need for intubation [4]. A CCB may improve pulmonary vasculature perfusion as it does so in other pulmonary conditions. 

**Figure 1:**
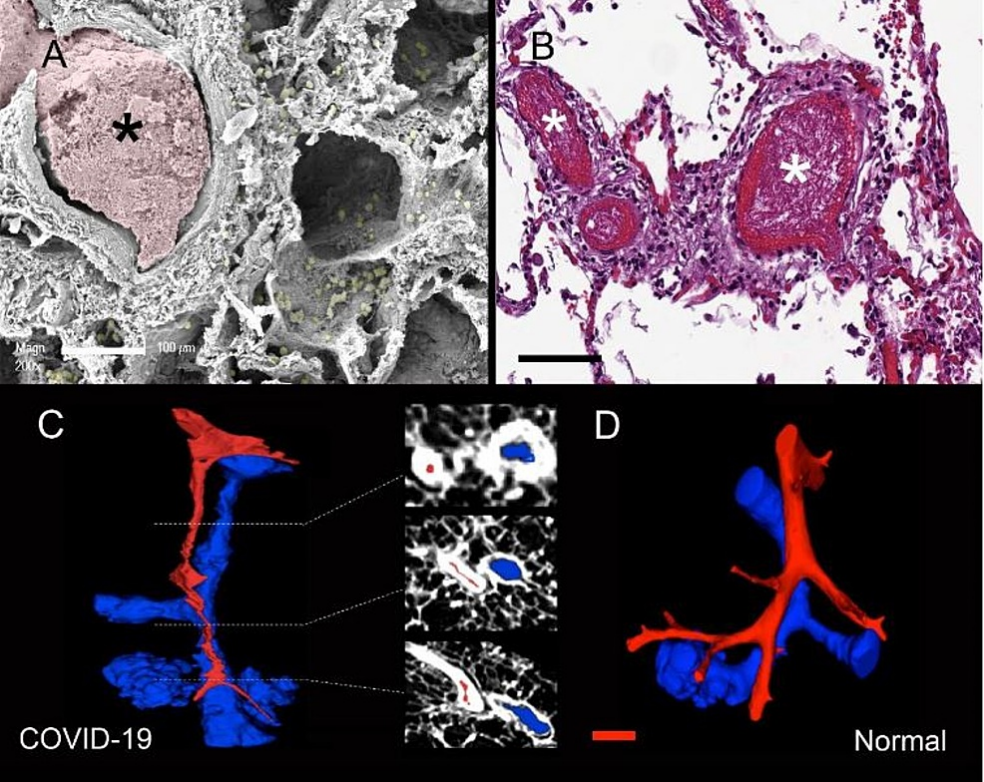
Micro-computerized tomography-based 3-dimensional reconstruction COVID-19-associated thrombosis. A, B: In the inflamed vessels, there were multifocal thrombi (*) with (sub) total vascular occlusion of both pulmonary arteries and veins as visualized by scanning electron microscopy (A) and conventional histopathology (B) (scale bar = 100um). In the scanned electron microscopy image, the thrombus is pseudocolored pink, and the infiltrating lymphocytes are pseudocolored yellow. C, D: μCT-based 3D reconstruction of subsegmental pulmonary arteries (red) and airways (blue) demonstrated (sub)total occlusion of the arteries in COVID-19-lungs (C), as compared to uninfected controls (D) (scale bar = 300 μm) [7]. Figure inserted with permission from publisher [7].

The pulmonary microvasculature is significantly affected by COVID-19 induced endothelial injury and hypoxia that can lead to intussusceptive changes [7]. Vasoconstriction is apparent on the micro-CT 3D reconstruction (Figure 1 ) of sub-segmental pulmonary arteries and airways [7]. 

Systemic pulmonary vasodilators can help alleviate vasoconstriction

**Figure 2:**
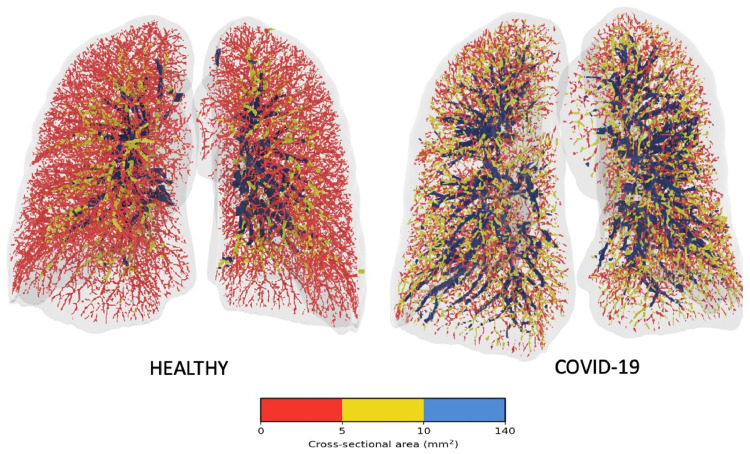
3D reconstructions of the pulmonary vasculature in a healthy patient and a COVID-19 patient Visual representation of the blood vessels is colored according to their size. Red denotes the small vessels, yellow the mid-size vessels, and blue indicates the larger vessels. Segments are color-coded according to size, and the relative and marked absence of small vessels (colored red) is notable as is the marked “proliferation” of medium-sized yellow vessels in the COVID-19 lungs vis-à-vis the healthy lungs. Figure inserted with permission from publisher [8].

In a study of over 100 high-resolution CT scans [8] a significant reduction in pulmonary blood volume contained in small blood vessels of <5 mm^2^ was found in patients with COVID-19 when compared with healthy volunteers. Moreover, this was found to clinically correlate with a need for more oxygen supplementation [9]. Three-dimensional reconstructions of pulmonary vasculature (Figure 2) illustrate a paucity of small vessels in lungs afflicted by COVID-19 in comparison to healthy lungs [8].

This paucity parallels a loss of blood volume in small vessels, as well as an increase in volume in medium to large vessels and, has been described as a “redistribution” of blood volume [8]. This may represent the backup of flow with proximal dilatation as a result of distal small vessel vasoconstriction. The importance of small or microvessel perfusion is essential for the maintenance of adequate oxygenation since that is precisely where gas exchange occurs. 

Perfusion defects in the context of ventilation-perfusion mismatch in COVID-19 have been investigated. Mathematical modeling of perfusion abnormalities in COVID-19 has implicated extensive perfusion defects, perfusion defects combined with ventilation-perfusion mismatching in the non-injured lung, and hyperperfusion with up to a threefold increase in perfusion of the afflicted regions [10]. Clinical findings are consistent as hypoperfusion was observed in areas of the healthy lung and increased blood flow or hyperperfusion to areas of the non-aerated lung [11]. The increased ventilation-perfusion ratio in areas of the healthy lung was attributed to pulmonary vasoconstriction [11].

**Figure 3:**
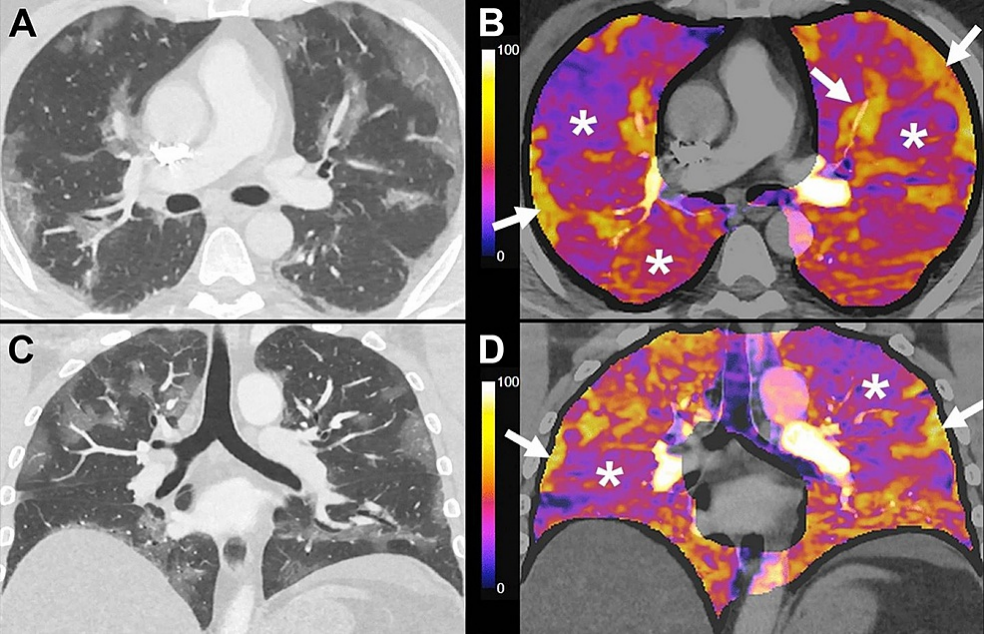
Perfusion CT imaging of a COVID-19 lung reveals areas of hyper and hypoperfusion The CT scan is of the lungs of a 37-year-old male patient with RT-PCR-confirmed COVID-19 and was taken 10 days post symptom onset. Admission PaO2/FiO2 ratio was 240 and the d-dimer level was 480 ng/mL. The patient was admitted to the ICU and managed with invasive mechanical ventilation. A, C: Axial and coronal lung-window chest CT angiography images show multiple foci of ground-glass opacities, with a predominantly subpleural distribution, with areas of apparently healthy lung parenchyma. Vascular dilatation can be seen in areas of ground-glass opacities. B, D: 5 mm reconstruction images of subtraction iodine maps in corresponding axial and coronal planes, show areas of severe hypoperfusion in the corresponding healthy lung parenchyma (*), with increased perfusion in areas of ground-glass opacities (white arrows). Figure inserted with permission from publisher [12].

In iodine-enhanced perfusion CT scans of over 40 patients, abnormalities were found in 87.8%, mainly hypoperfusion in areas of the apparently healthy lung [12]. That same study found stark imaging features (Figure 3) of increased perfusion to areas of ground-glass opacities [12].

This aspect of having less perfusion to the healthy lung and increased perfusion to the injured lung is an important one to consider. Especially as it sabotages ventilation-perfusion matching and is a recipe for compromised oxygenation. Moreover, patients with severe hypoperfusion in areas of apparently healthy lung parenchyma had an increased probability of being admitted to the ICU and initiated on invasive mechanical ventilation [12]. This is resonant with the study cited earlier that showed decreased risk for mechanical ventilation in patients that were on vasodilatory medication [4]. Although hyperperfusion may occur in some areas of the lung, hypoperfusion manifests in others. What matters most are the clinical outcomes that follow. This includes actual net-sum hypoxia, overall pulmonary function, and ultimately survival.

## Discussion

Reversing hypoperfusion of otherwise amenable healthy segments can provide a pathway to counteract hypoxic development and disease progression. Offsetting vasoconstriction and promoting perfusion in the healthy lung can be achieved with vasodilation agents. The cumulative effect of improved perfusion with vasodilation may be responsible for improved oxygenation. Moreover, when a diffuse bilateral process involves multiple lobes, a paralleled counteractive response with systemic intravascular vasodilation may be necessary. Ultimately this could be reflected in the positive outcomes of survival. Ultimately, this may be reflected in the positive outcomes of survival observed in the several studies of CCB therapy mentioned above.

Furthermore, the endothelium plays an essential role in vasomotion [13] and hypoxic pulmonary vasoconstriction [14]. In COVID-19, endothelial inflammation is a reality [15]. Correspondingly, pulmonary vascular endothelialitis and alveolar-capillary thrombosis are critical elements of pathogenesis in COVID-19 [7]. Many patients develop microthrombosis following prolonged perfusion deficits from the disease [16]. This has led to the utilization of anticoagulation to improve survival in hospitalized patients with COVID-19 [17]. The latter helps preclude the development of microthrombosis or micro-clots in the pulmonary circulation. Beenen et al. [16] broaden the perspective on how perfusion defects play a critical role:* *

"In conclusion, invasively mechanically ventilated ICU patients with severe COVID-19 not only can develop pulmonary embolism but also show large scattered areas of severely diminished perfusion consistent with diffuse pulmonary microcirculatory dysfunction. These defects seem to be independent of the presence of pulmonary embolism, possibly reflective of microthrombi in the pulmonary circulation. The combination of extensive parenchymal involvement with diffuse perfusion abnormalities may explain the occurrence of severe and persistent respiratory failure that is frequently seen in patients with severe COVID-19 pneumonia who require mechanical ventilation."

Altogether, clot formation may be closely related to the impedance of blood flow in the setting of endothelial injury and inflammation. Indeed, Virchow’s triad is evoked.

However, the attention placed on ameliorating perfusion defects related to vasoconstrictive development at the outset has been wanting. Disease-ridden oxygenation in COVID-19 may potentially be reclaimed, not by exhausting ventilation modalities but rather by relieving perfusion defects via the promotion of pulmonary vasodilation.elax airways, systemic pulmonary vasodilators may help alleviate vasoconstriction. This can serve to render measures provided to support ventilation, including bronchodilators more capable of being effective in improving oxygenation. While bronchodilators relax airways, systemic pulmonary vasodilators may help alleviate vasoconstriction. Both can work in tandem to synergistically improve outcomes in COVID-19.

Sustaining both satisfactory ventilation and perfusion is essential to improve oxygenation. They are two sides of the same coin. Perfusion abnormalities are an integral aspect of hypoxia in COVID-19 in that oxygenation has been proposed to improve by decreasing impedance and improving flow via the alveolar-capillary unit [4]. In other words, an impedance of perfusion triggered by an endothelial injury can be counteracted with vasodilatory medication. Increased blood flow via the alveolar-capillary unit can enhance oxygen-carrying capacity.

This viral illness is characterized by a slow deteriorating course. In the same study that found severe endothelial injury, alveolar-capillary microthrombi and angiogenesis - intussusceptive changes increased with time and the number of days hospitalized [7]. This indicated a gradual yet progressive process. Therefore, consistently maintaining patent perfusion over the entire course of hospitalization can help patients emerge from the disease and survive i.e., over approximately 10 to 14 days, at least, to encompass a significant duration, if not the entire duration of the illness.This is without expecting a rapid improvement. Rather, maintaining vasodilating therapy temporally matched with the virally inflicted inflammation can help ameliorate the gradual deleterious progression of the disease. Vasoconstriction associated with endothelial inflammation may be mitigated with this method. Maintaining persistent patency of perfusion can allow for a negotiated improvement. This may partially explain the benefits of CCB therapy in that consistent vasodilation maintained in the hospital as part of an antihypertensive regimen may have contributed toward survival.

Exploration of other pulmonary vasodilators in the treatment regimens of COVID-19 may be beneficial. Moreover, factors that play a role in vasomotor responses can be appreciated. From this perspective, vasoactivity related to hypoxia may be better understood in the context of various clinical conditions.

For example, iron deficiency is by itself a risk factor for mortality in COVID-19 [18]. Although iron has myriad effects, it also alters pulmonary vascular responses to hypoxic exposure. Especially since increases in pulmonary artery pressures caused by hypoxia depend on iron status [19]. In addition, intravenous therapy prevented increases in pulmonary arterial systolic pressure [19]. Correspondingly, hypoferremia was found to predict hospitalization and oxygen demand in COVID-19 patients [20]. Serum iron levels are lower in patients with high oxygen demand already at the time of admission [20]. Reduced iron availability is also associated with and may even contribute to the progression of acute respiratory distress syndrome (ARDS) in COVID-19 patients [20].

Furthermore, vitamin D also correlates with pulmonary hypertension (PH) as systolic pulmonary artery pressures of patients with low vitamin D were found to be higher than in control groups [21]. Low plasma 25-hydroxyvitamin D [25(OH) D] levels also appear to be an independent risk factor for COVID‐19 infection and hospitalization [22]. Moreover, vitamin D replacement in patients with pulmonary arterial hypertension resulted in the significant improvement of right ventricular size and six-minute walk testing, although mean pulmonary artery pressure improved nonsignificantly [23]. Impaired right ventricle function is associated with mortality in COVID-19 infection as well [24]. The right ventricular function may trail the “redistribution” and backup of blood volume with enlargement of medium to large vessels and losses in small vessels as mentioned above. Vitamin D replacement functions in various ways, nonetheless, considering vasoactive responses of the pulmonary vasculature may be another aspect that is worthy of attention.

Other vasodilators such as sildenafil have also been suggested for use and studied in COVID-19. A specialized cardiorespiratory team in the United Kingdom utilized sildenafil as a first-line agent after identifying that 84% of patients had PH [25]. This was evidenced by echocardiography showing right ventricular dilatation and/or raised pulmonary artery systolic pressure. In that study, 52% of patients also developed pulmonary thrombosis. The noted findings of this expert team provide the following insight:

"We were encouraged by the use of sildenafil in our patients with pulmonary thrombosis and PH. The concept of sildenafil use was based on experience with managing patients with pulmonary thrombosis and PH in the non-COVID-19 setting. We also believed that this agent could be particularly useful in vasodilating peripheral pulmonary vessels. An improvement in echocardiography assessment was observed in all patients who received sildenafil and we did not encounter side effects. We believe that further studies are required to define the role of sildenafil in COVID-19 patients with PH whether or not this is associated with pulmonary thrombosis [25]."

Furthermore, in a triple-blind placebo-controlled trial, sildenafil was found to decrease the need for the initiation of invasive mechanical ventilation and reduced the length of stay in the hospital [26].

Utilization of vasodilatory medications such as phosphodiesterase inhibitors as well as CCBs among others can alleviate vasoconstriction, augment perfusion along the pulmonary vasculature and allow for improved oxygenation. Inhaled nitric oxide is another vasodilator that may provide benefits as well. Yet, given the cost, access, and ease of use, other medications may be more practical. Moreover, the consistent treatment aimed at countering vasoconstriction over a significant portion, if not the entire course, of the illness may make it difficult to provide nitric oxide to meet the ongoing need that can last longer than 10 days at times. That being said, if these hurdles can be overcome, then evaluating the efficacy of intravascular medication versus inhaled regimens and perhaps both may be considered. The perfusion patterns outlined above are important aspects to take into account. Theoretically, correcting vasoconstriction in the healthy lung with vasodilation via an inhaled agent seems appropriate. But the discreetness between the healthy lung and diseased lung is not fixed. With time, the disease progresses and more of the lung is involved. The transition between the two is a process. Mitigating perfusion defects from the outset with non-discriminant vasodilation could be most beneficial in the long term as reflected by the clinical studies listed above.

Aberrant perfusion patterns affecting multiple lobes of bilateral lungs represent a diffuse process that may best be corrected by intravascular medications that are delivered extensively. But further research including physiologic models may provide further insight into this matter. Clinically, it was already observed that long-acting amlodipine was found to have improved survival even better than other CCBs including nifedipine [2]. This hearkens back to the point made earlier - that consistent perfusion maintenance may help mitigate burden over the long-term disease course and thereby improve survival. Along this line of approach, perhaps the study of tadalafil, a long-acting phosphodiesterase inhibitor, could be found to be even more effective with less of an impact on blood pressure that can render it amenable for patients without hypertension as well.

## Conclusions

The central goal is to promote perfusion along the alveolar-capillary unit as part of a strategy to improve oxygenation. With perfusion deficits that persist and progress, ventilatory supportive measures can be insufficient to overcome an underlying lack of perfusion to viable healthy tissue. Therefore systemic pulmonary vasodilation should be pursued. Combining this with steroids and anticoagulation may each contribute towards this objective. Various pulmonary vasodilator agents may also be investigated as part of therapeutic approaches to mitigate and improve outcomes and survival in this disease. Improved outcomes that include survival have been observed in clinical studies of calcium channel blockers in general and in amlodipine in particular. Sildenafil has also been found to decrease the need for mechanical ventilation as well as reduce the length of hospital stay. Promoting vasodilation may provide benefit via an approach that is independent of and perhaps complementary to other antiviral and immunomodulatory agents that are already available. Ultimately, vasodilatory agents may enhance vital outcomes in this disease.
